# High-grade endometrial stromal sarcoma presenting in a 28-year-old woman during pregnancy: a case report

**DOI:** 10.1186/1752-1947-4-243

**Published:** 2010-08-04

**Authors:** Frédéric Amant, Kristel Van Calsteren, Maria Debiec-Rychter, Liesbeth Heyns, Katya Op De Beeck, Xavier Sagaert, Bart Bollen, Ignace Vergote

**Affiliations:** 1 Gynecologic Oncology, Leuven Cancer Institute (LKI), Katholieke Universiteit Leuven, Belgium; 2 Center for Human Genetics, Katholieke Universiteit Leuven, Belgium; 3 Department of Radiology, Katholieke Universiteit Leuven, Belgium; 4 Department of Pathology, Katholieke Universiteit Leuven, Belgium; 5 Obstetrics and Gynecology, Maria Hospital Overpelt, Belgium

## Abstract

**Introduction:**

To the best of our knowledge, soft tissue sarcomas have not prevously been reported as a complication during pregnancy.

**Case presentation:**

A 28-year-old Caucasian woman was diagnosed with a transperitoneal sarcoma during pregnancy. Morphological, immunohistochemical, chromosomal and mutational analyses pointed towards a high-grade endometrial stromal sarcoma. Although surgery and chemotherapy are possible during pregnancy, we were unable to perform these in this case.

**Conclusion:**

The potential to treat gynecological cancer during pregnancy should always be assessed individually.

## Introduction

Recent literature shows an increased interest in cancer complicating pregnancy. This is a result of the realization that oncological treatment modalities, including surgery and chemotherapy, can be applied after the first gestational trimester without hampering the fetus [1,2].

Evidence from western countries shows that mainly breast cancer and hematological malignancies are diagnosed during pregnancy [3]. Gynecological cancers also significantly contribute to the problem. Cancer of the cervix is the second most common cancer among women worldwide and the most common gynecological cancer in the developing world [4]. Incidence rates of cancer complicating pregnancy therefore vary around the world. Especially with this perspective in mind, guidelines for the treatment of gynaecological cancer were recently proposed [5]. In contrast, sarcomas are uncommon and increase with age. Apart from bone sarcomas, we are not aware of other sarcomas complicating pregnancy. Here, we describe a fatal case of a high-grade endometrial stromal sarcoma (ESS) diagnosed at a gestational age of 19 weeks.

### Case presentation

A 28-year-old Caucasian woman consulted her gynecologist with pain in the right fossa at a gestational age of 15 weeks. Her medical history was straightforward. She smoked 10 cigarettes per day for more than 10 years. Sonographic examination suggested an appendicular plastron and was interpreted as an ovarian mass. Subsequently, a laparoscopy was performed in a district hospital. Due to the pregnancy and the adhesions the view was incomplete (the uterus and ovaries could not clearly be identified) but peritoneal spread of malignant plaques was evident. Microscopic examination of the peritoneal lesions showed a solid, fat-infiltrating mass, composed of cancerous cells with storiform growth pattern. Cancer cells have a spindle form containing a moderate quantity of eosinofilic cytoplasm and a polymorph vesicular nucleus, sometimes containing a prominent nucleolus. More than 10 mitotic figures per 10 high-power fields were present, including abnormal mitotic figures. This morphology corresponds to a high grade sarcoma. Immunohistochemistry was performed and the tumor cells revealed the following immunophenotype: desmin (-), alpha SMA (+++), CK7 (+), CK20 (-), CD117/C-Kit (-), S100 (-), CD34 (-), C125 (-), EMA (-), CD10 (diffuse +++), calretinine (-), CK 5.6 (-), MDM 2 (-), ER (-), PR (-). The positive staining for CD10 and alpha-smooth muscle actin (alpha-SMA) in the absence of desmin expression may be indicative for a sarcoma of endometrial stromal origin.

Chromosome preparations from the tumor specimen were obtained using standard primary culture procedures. For diagnostic purposes the karyotype was determined:

66-71<3N>,XXX,+X,-1,der(2)t(1;2)(p35;q37)),-7,+11,-13,-14,der(14;15)(q10;q10),-15,-16,+17,der(18)t(7;18)(q11;q23),+20,+21,+21 [cp17]. Hence, the tumor karyotype was not specific for any known translocation-related or other sarcomas.

In order to exclude the possibility of KIT-immunonegative gastrointestinal stromal tumor, mutational analyses were performed using a combination of polymerase chain reaction (PCR) amplification, denaturing high-performance liquid chromatography (D-HPLC) pre-screening, and bi-directional sequencing, as described previously [6]. Tumor specimen showed wild-type genotype for exons 9, 11, 13, 17 of the *KIT *or exons 12, 14 and 18 of *PDGFRA *genes. Thus, the mutational analysis was not indicative for any particular sarcoma. Therefore, the final diagnosis was most suggestive for high-grade ESS.

**Figure 1:**
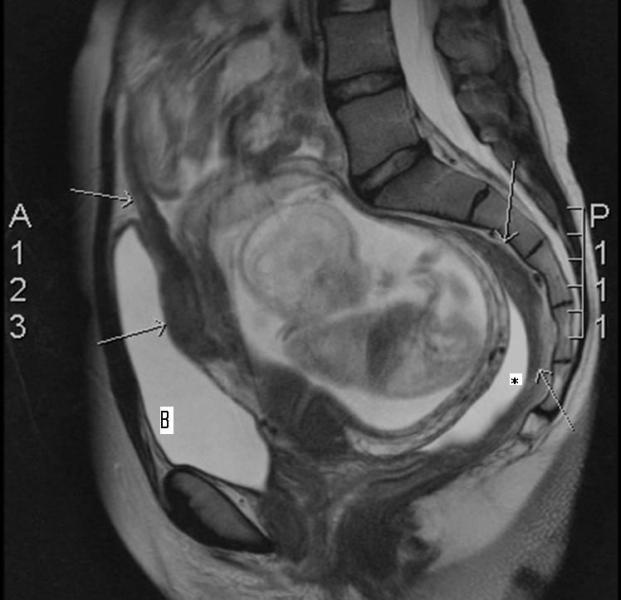
**Magnetic resonance imaging findings of diffuse peritoneal involvement by a poorly differentiated sarcoma**. Sagittal T2-weighted turbo spin-echo magnetic resonance image (repetition time msec/echo time msec = 8440/136) shows diffuse sheetlike and nodular thickening of the peritoneal surfaces (arrows). Note also a moderate amount of ascites (asterisk). Bladder (B).

Subsequently, she was transferred to our hospital. Magnetic resonance imaging showed diffuse peritoneal and omental tumoral implants, spreading along the visceral surfaces of the small bowel and large bowel, without a definable primary mass (Figure 1). Also a moderate amount of ascites was present. There were no signs of hepatic and lymph node metastasis. Computer tomography of the lungs excluded metastasis.

**Figure 2:**
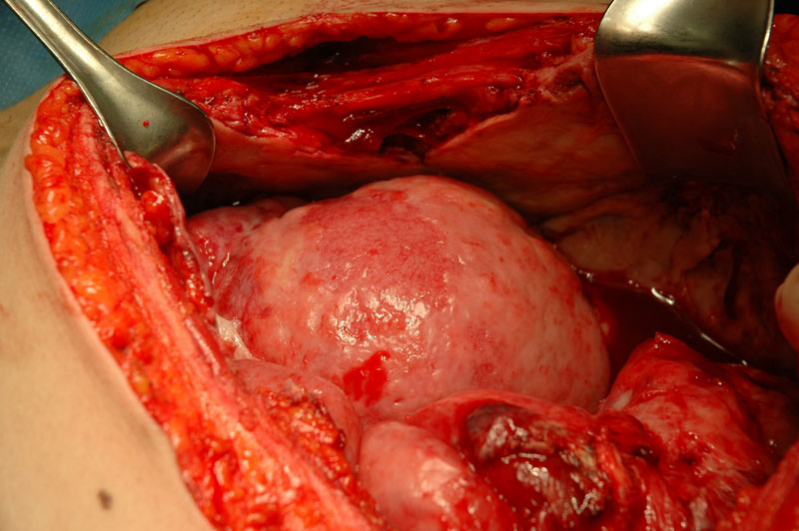
**Peroperative findings indicating diffuse tumoral infiltration of the uterine serosa**.

We discussed the diagnosis of a high-grade ESS with transperitoneal spread, but without distant metastasis, with the patient and her husband. Psychological support was provided. We explained that the situation was life threatening for both the mother and fetus. Given the young age of the patient and expected limited response to chemotherapy, we opted for a maximal surgical effort during cytoreductive surgery. If this had been a case of a significant cytoreduction, we would have considered anthracyclin based chemotherapy, even in the presence of an ongoing pregnancy. We agreed that if the maternal situation seemed prospectless, termination of pregnancy should be performed. In which case, hysterotomy would appear to be a better solution when compared to induction and labor. At midline laparotomy, the tumor was diffusely spread throughout the pelvis and upper abdomen. The disease at the level of the peritoneum was infiltrating the sub-peritoneal fat and this infiltration was responsible for the pain at the right fossa. The uterine serosa was diffusely involved (Figure 2). The small and large bowel and the omentum contained diffuse and multiple tumoral plaques. Given the diffuse and sometimes deep infiltration of both the peritoneum and intestines, she was considered inoperable. A hysterotomy was performed, leaving the uterus *in situ*. The placenta was macroscopically and microscopically normal. Three days later, intestinal obstruction was diagnosed. We agreed that chemotherapy was not likely to be a clinical benefit for a high-grade sarcoma causing intestinal obstruction whereas the potential for sepsis was considerable. Symptomatic treatment was initiated. She died at home six weeks after diagnosis.

## Discussion

To the best of our knowledge, this is the first case of a transperitoneal high-grade ESS complicating pregnancy. Despite our policy to explore all possibilities in order to maintain the pregnancy, we were unable to save the fetus.

After diagnostic work-up, we agreed that the tumor resembled a high-grade ESS. However, this designation should be used cautiously. Most previously so-called high-grade tumors lack the typical growth pattern and vascularity of low-grade ESS and show destructive myometrial invasion rather than the lymphatic permeation of a low-grade ESS. Moreover, they demonstrate marked cellular pleomorphism and brisk mitotic activity. Tumours that used to be termed high-grade ESS are currently called poorly differentiated or undifferentiated uterine sarcoma [7,8]. Occasional tumors as the one described here have been reported that are high-grade and of endometrial stromal derivation [7]. Although we were unable to examine the uterus and confirm this diagnosis, the combination of morphological, immunohistochemical, chromosomal and a mutational analysis suggests high-grade ESS. We emphasise that some would call this an undifferentiated sarcoma. Based on the absence of hormone receptors, we do not believe that hormonal stimulation during pregnancy has a role in the origin of the sarcoma. Cancers complicating pregnancy reflect the young age of the mother rather than an etiologic role of pregnancy.

In order to treat the patient and preserve the pregnancy, we considered major surgery and chemotherapy. Laparoscopy and explorative surgery were performed in this patient. Laparoscopy can be performed safely in experienced hands and has the same advantages as in non-pregnant women [9-11]. The carbon dioxide pneumoperitoneum and carbon monoxide production during electro-coagulation seems not to be hazardous to the fetus as long as the maximal pressure (13-15 mmHg) and operation time (25-90 minutes) are respected. Open laparoscopy (opening of the peritoneum under direct visualisation instead of using the Verres-needle) is advised in order to avoid uterine perforation. Abdominal surgery can be performed safely during pregnancy if physiologic adaptations are considered and the patient is monitored adequately, preventing hypoxia, hypotension and hypoglycemia [12]. Outcome data described in literature suggest there is no increased risk of miscarriage and congenital anomalies. Only in cases of peritonitis is the fetal loss rate increased [13]. Apart from urgent surgery, including appendectomy and cholecystectomy, oncological surgery can also be performed. We based our decision to attempt to cytoreduce the patient on previous successful experience including debulking surgery with preservation of the pregnancy for advanced stage ovarian cancer [14,15].

Chemotherapy can be administered in the second and third trimester of pregnancy, after organogenesis [1]. Anthracyclines have a particular efficacy against sarcomas. From previous experience in breast cancer and hematological malignancies occurring during pregnancy, there is considerable evidence on the safety of anthracyclines on the fetus [1,2].

We opted for an exploratory laparotomy to remove the tumor. However, the operative findings proved untenable given the diffuse and deep infiltration of the abdominal wall and small bowel and colon. The decision to terminate the pregnancy was based on the extensive transperitoneal spread of a high-grade sarcoma, the limited sensitivity of sarcomas to cytotoxic drugs and the diffuse uterine involvement. This situation would not allow a pregnancy to develop. This option was discussed preoperatively with the parents and allowed us to surgically remove the pregnancy by hysterotomy rather than bring her to labor ward for a prostaglandin induction.

## Conclusion

This case shows that loss of pregnancy may be inevitable, despite the theoretical potential to perform major surgery and to administer chemotherapy during pregnancy. The treatment of gynecological cancer during pregnancy is case dependent.

## Abbreviations

Alpha-SMA: alpha-smooth muscle actin; DHPLC: denaturing high-performance lquid chromatography; ESS: endometrial stromal sarcoma; PCR: polymerase chain reaction.

## Consent

Written informed consent was obtained from the patient's next of kin for publication of this case report and accompanying images. A copy of the written consent is available for review by the journal's Editor-in-Chief.

## Competing interests

The authors declare that they have no competing interests.

## Authors' contributions

The manuscript was written by FA, KVC and LH. MDR performed the genetic analysis; KODB provided the MRI images; XS was responsible for the pathological examination. BB, FA and IV were involved in the diagnosis and treatment of the patient. All authors provided review and editing of the manuscript. All authors read and approved the final manuscript.

## Authors' information

FA is Senior Clinical Investigator for the Research Fund-Flanders (Belgium) and KVC is Researcher for the Research Fund-Flanders (Belgium).
